# Continuation and discontinuation of antidepressant treatment before, during and after pregnancy: a cohort study

**DOI:** 10.1007/s00737-026-01683-5

**Published:** 2026-03-10

**Authors:** Ella J. Marson, Irene Petersen, Sonia Saxena, Patricia Schartau, Holly Christina Smith

**Affiliations:** 1 UCL Research Department of Primary Care and Population Health, London, UK; 2 Primary Care Imperial College School of Public Health, London, UK

**Keywords:** Electronic health records, Perinatal depression, Epidemiology

## Abstract

**Objective:**

To assess the relationship between antidepressant treatment before, during and after childbirth.

**Design:**

Cohort study.

**Setting:**

Primary care in the United Kingdom (UK).

**Participants:**

Women aged 15–49 with a single live birth (2006–2015), stratified by antidepressant history:

- **Cohort A**: Prescribed antidepressants ≥ 2 years before childbirth.
- **Cohort B**: Prescribed 1–2 years before childbirth.
- **Cohort C**: Prescribed within 1 year before childbirth.
- **Cohort D**: No antidepressants prescribed before childbirth.

**Outcome measures:**

Changes in antidepressant treatment during the perinatal period.

**Results:**

Of 202,303 women, 46,479 (23%) had pre-childbirth antidepressant prescriptions (Cohorts A: 37,161; B: 19,627; C: 14,363), while 155,824 (77%) did not (Cohort D). In cohort A, 76% discontinued treatment in the year before childbirth, compared to 48% in cohort B. Postpartum, 22% of those in cohort A and 33% in cohort B restarted treatment. Overall, 26,835/202,303 (13%) received antidepressants in the year after childbirth. Among women without prior treatment, 10,258/155,824 (7%) started antidepressants postpartum, compared to 16,577/46,479 (36%) of those with prior use. Cohort C had the highest postpartum prescription rate (9,232/14,363, 64%).

**Conclusions:**

Antidepressant treatment after childbirth is common, particularly among women who have received treatment before childbirth. Many women discontinue antidepressants before or during pregnancy. The study highlights the importance of considering history of antidepressant treatment when planning postnatal care and counselling women on continuing or stopping antidepressants during pregnancy. This information can aid healthcare professionals in advising women about antidepressant use before, during, and after pregnancy, considering individual circumstances and risks.

**Supplementary Information:**

The online version contains supplementary material available at 10.1007/s00737-026-01683-5.

## Article highlights

Previous studies typically have small cohorts and do not describe the changes in antidepressant treatment before pregnancy, during pregnancy and following childbirth.

We found that half of those with a previous prescription discontinued treatment during conception/pregnancy, but a third of these restart treatment in the year after childbirth.

Of those with no previous antidepressant treatment, 7% were issued a prescription in the year after childbirth compared to 36% of those with any prior treatment.

Nearly 2/3 of those who received treatment during conception/pregnancy were issued an antidepressant prescription in the year after childbirth.

**Supplementary Information:**

The online version contains supplementary material available at 10.1007/s00737-026-01683-5.

## Introduction

Perinatal depression refers to depression occurring during pregnancy and the postnatal period which is defined as up to a year after childbirth (Perinatal Depression n.d.). It is becoming increasingly prevalent, with a global estimate indicating that approximately 26% of women experience perinatal depression (Al-abri et al. 2023). The impact of perinatal depression can be profound, affecting both mother and child, sometimes with consequences that extend beyond the postnatal period (Netsi et al. 2018, Shen et al. 2016, Field 2010). The exact causes of perinatal depression are not fully understood but are believed to stem from a complex interaction of hormonal fluctuations, genetic predispositions, and social factors, such as the level of social support after childbirth and housing conditions (Stewart Donna 2016, Bloch et al. 2003). One of the strongest predictors of perinatal depression is a previous history of depression (Al-abri et al. 2023, Davé et al. 2010, Boyce 2003, Silverman et al. 2017). 

While antidepressants are an effective treatment for perinatal depression (Depression n.d.), many women discontinue treatment during pregnancy. Around 3% of women of childbearing age require antidepressant treatment (McCrea et al. 2016)​, yet many discontinue their medication during pregnancy due to concerns about potential risks to the fetus (Trinh et al. 2023, Trinh et al. 2022, Hung et al. 2023, Wikman et al. 2020, Petersen et al. 2011). A systematic review examining the safety of antidepressants during pregnancy found that, while there is some evidence of an increased risk for adverse outcomes, the absolute risks remain small, and adverse outcomes are rare (Desaunay et al. 2023). The lack of randomized controlled trials in this area introduces a possibility of confounding and biases impacting these findings (Desaunay et al. 2023). Furthermore, discontinuing antidepressants during pregnancy can result in relapse of depression, which poses significant risks for both the mother and fetus, including the potential for withdrawal symptoms (Liu et al. 2022). Far less is known about antidepressant discontinuation before conception, but evidence suggests that stopping treatment in the pre-pregnancy period may also increase the risk of relapse (Cohen et al. 2006, Liu et al. 2022).

Understanding the patterns of antidepressant treatment before, during, and after pregnancy in a large cohort from Primary Care settings in the UK can offer insights into how the treatments are managed during the perinatal period. Identifying patterns of continuation and discontinuation of antidepressant use can help healthcare professionals identify women at higher risk of postnatal depression, enabling better-targeted interventions.

## Aim

The aim of this study was to examine the relationship between antidepressant treatment before and during pregnancy and the likelihood of antidepressant treatment after childbirth.

## Methods

### UK healthcare

In the UK, healthcare is free at the point of delivery for all residents as part of the National Health Service (NHS). Primary care is typically the first point of contact and is largely delivered by General Practitioners (GPs) and other healthcare professionals (nurses and health visitors) within a practice. Information about patients and their health are collected during primary care consultations and entered on the general practice computer system. This information is primarily used for clinical care but is also widely used for research through large healthcare databases.

### Data source

We used IQVIA Medical Research Database (IMRD), one of the largest UK primary care electronic health record (EHR) databases. The database contains anonymised patient-level information on demographics, prescribing, symptoms, procedures, prevention, lifestyle factors and diagnostics. Socioeconomic information is captured through Townsend score, which provides a measure of material deprivation based on where a person lives, unemployment, car ownership, home ownership and household overcrowding (Health, Deprivation P, Townsend P, Phillimore A Beattie Health and Deprivation n.d.). As of December 2016, IMRD contained anonymised electronic health records for 16 million registered patients from 730 practices across the UK. The database is broadly representative of the UK population in terms of demographics, chronic disease and mortality; however, there is an over-representation of more affluent people (Blak et al. 2011).

Ethical approval was granted by the IQVIA Scientific Review Committee on 28/08/2020 and 10/04/2019 for Scientific Review Committee protocol number: 20SRC052 and Protocol number: 19THIN013 respectively. IQVIA Medical Research Data (IMRD) incorporates data from THIN (previously The Health Improvement Network), a Cegedim Database. Reference made to IMRD is intended to be descriptive of the data asset licensed by IQVIA.

### Study design

A cohort study investigating antidepressant treatment before, during and after pregnancy.

### Participants

We drew on a preexisting cohort of women who had a record of a single live birth (Smith 2023, Petersen et al. 2016). From this, all women aged 15 to 49 years old who had a single live birth between 1 st January 2006 and 31 st December 2015 were eligible for inclusion in this study. Exclusion criteria included women who had been registered at a GP practice for less than six months before date of childbirth; missing social deprivation information (Townsend scores) and those with incomplete follow-up for one year after childbirth (due to transferring practice or death). When a woman had more than one eligible pregnancy within the study period, one pregnancy was chosen at random to be included in the study.

### Antidepressant treatment

We considered women to be on antidepressant treatment if they were prescribed at least one antidepressant. Any antidepressant prescriptions issued to women from study start date (1st January 2006 or when the women registered with the GP) to one year after the date of childbirth were captured from their electronic records. Antidepressant prescriptions included selective serotonin reuptake inhibitors, tricyclic antidepressants, monoamine oxidase inhibitors and other antidepressant drugs. However, any prescriptions for amitriptyline or duloxetine were excluded as most likely related to analgesia or anxiolytic treatment rather than depression. The women were then assigned to one or more cohorts based on the timings of their antidepressant prescriptions – Cohort A: included women who received antidepressant prescriptions issued two or more years before childbirth, Cohort B: included women who received antidepressant prescriptions which were issued at any time from two years to one year prior to childbirth, Cohort C: included antidepressant prescriptions issued at any time in the one year before childbirth. It has been suggested that women may stop their antidepressants before trying to conceive so one year prior to childbirth was chosen to incorporate conception and pregnancy (McCrea et al. 2016). Thus, women who received treatment would be included in one or more of the three cohorts. Those without an antidepressant prescription prior to childbirth were categorised as having ‘no previous history’. Women were followed up for one year after childbirth to capture treatment after childbirth.

### Maternal characteristics

Our analyses were stratified by antidepressant treatment prior to childbirth and by maternal age at childbirth, child’s year of birth, and socio-economic status (Townsend score). Women were assigned to a 5-year band according to their age. We used Townsend score fifths whereby each woman is assigned to one of five groups of deprivation, from least to most deprived. Year of childbirth was grouped into 2-year bands.

### Statistical analysis

A table summarised women’s characteristics at childbirth, stratified by antidepressant treatment status and the timing of their most recent prescription. We developed logistic regression models to explore the likelihood of having an antidepressant treatment in the year after childbirth. These models were also stratified by prior treatment and by maternal age, social deprivation and calendar year. Adjusted models, accounting for maternal age, social deprivation and calendar year are also presented. A random-effects term (random slope and random intercept) was used in all models to account for possible clustering by GP practice. Odds ratios and 95% confidence intervals were reported. Log Likelihood Ratio tests were performed to evaluate whether there was an interaction between age and socio-economic status (Townsend score).

Sankey diagrams were generated to illustrate the changes in antidepressant treatment from pre-conception, through to pregnancy and after childbirth. Sankey diagrams provide a visual representation of the movement of patients from one point to another, and the width of the arrows are proportional to the flow of the patients between points.

All analysis was conducted using Stata, version 17.

### Patient and public involvement

We are very grateful to members of the public who generously gave their time to feedback on the conception of this study, in particular we are grateful to the many women who have shared their experiences of childbirth and beyond, and who serve as a continued source of inspiration for this research.

## Results

We identified a total of 438,538 pregnancies and childbirths. After applying the study’s inclusion and exclusion criteria, a final sample of 202,303 were included in the study (see Supplementary Figure A).

### Antidepressant treatment before childbirth

Overall, 23% (46,479/202,303) of women had received at least one antidepressant prescription at some point before childbirth (Table 1). This proportion was highest among women aged 25–29 years, 40–44 years, and 45–49 years (26–28%) and lowest among women aged 15–19 years (9%). The prevalence of antidepressant treatment increased with socioeconomic deprivation, from 18% (7,790/43,405) in the least deprived areas to 29% (9,334/31,746) in the most deprived areas (Table 1). The proportion of women receiving treatment increased slightly (from 29% in 2006–2007 to 32% in 2014–2015) over time (Table 1).

**Table 1:** Baseline characteristics of women at time of childbirth, stratified by history of antidepressant treatment; and odds of antidepressant prescriptions after childbirth, stratified by previous prescribing history

Characteristic	Total	Individuals on no antidepressant prescription from study entry to childbirth	%	Individuals with at least one prescription from study entry to childbirth	%	Number of women receiving an antidepressant prescription stratified by timings						Odds of antidepressant prescription after childbirth		
						Cohort A	%**	Cohort B	%**	Cohort C	%**	All women adjusted^†^ OR (95% CI)	No prescriptions before childbirth adjusted^†^ OR (95% CI)	Prescriptions before childbirth adjusted^†^ OR (95% CI)
Total	202,303	155,824	77	46,479	23	37,161	80	19,967	43	14,363	31			
Maternal age														
15–19	6,327	5,760	91	567	9	174	31	294	52	304	54	1.82 (1.68–1.98)	3.94 (3.59–4.31)	1.64 (1.36–1.97)
20–24	27,012	20,209	75	6,803	25	4,308	63	3,475	51	2,529	37	1.65 (1.57–1.75)	2.46 (2.30–2.63)	1.18 (1.12–1.36)
25–29	49,039	36,435	74	12,604	26	10,098	80	5,326	42	3,684	29	1.23 (1.18–1.29)	1.35 (1.27–1.43)	1.02 (0.97–1.07)
30–34	63,397	49,947	79	13,450	21	11,294	84	5,565	41	3,963	29	1	1	1
35–39	44,223	34,397	78	9,826	22	8,458	86	4,027	41	2,858	29	1.01 (0.97–1.05)	0.90 (0.84–0.96)	1.01 (0.95–1.06)
40–44	11,650	8,604	74	3,046	26	2,660	87	1,207	40	965	32	1.11 (1.04–1.17)	0.89 (0.80–0.99)	0.97 (0.89–1.06)
45–49	655	472	72	183	28	169	92	73	40	60	33	0.98 (0.77–1.27)	0.73 (0.45–1.18)	0.81 (0.59–1.11)
Townsend Score quintile														
1 (least deprived)	43,405	35,615	82	7,790	18	6,319	81	3,224	41	2,246	29	1	1	1
2	39,615	31,694	80	7,921	20	6,427	81	3,271	41	2,325	29	1.13 (1.07–1.19)	1.08 (1.01–1.15)	1.05 (0.98–1.13)
3	45,207	34,933	77	10,274	23	8,228	80	4,365	42	3,108	30	1.24 (1.17–1.31)	1.12 (1.03–1.20)	1.06 (0.99–1.13)
4	42,330	31,170	74	11,160	26	8,847	79	4,867	44	3,467	31	1.44 (1.34–1.54)	1.20 (1.11–1.31)	1.11 (1.03–1.19)
5 (most deprived)	31,746	22,412	71	9,334	29	7,340	79	4,240	45	3,217	34	1.64 (1.50–1.79)	1.28 (1.16–1.42)	1.20 (1.12–1.29)
Year group of childbirth														
2006–2007	22,259	17,298	78	4,961	29	4,005	79	2,085	42	1,337	27	1	1	1
2008–2009	44,761	35,089	78	9,672	28	7,750	79	3,987	41	2,689	28	0.92 (0.87–0.96)	0.91 (0.84–0.98)	0.95 (0.88–1.02)
2010–2011	42,097	32,702	78	9,395	29	7,567	79	3,885	41	2,759	29	0.96 (0.91–1.01)	0.93 (0.86–1.00.86.00)	1.00 (0.93–1.07)
2012–2013	41,344	31,468	76	9,876	31	7,833	79	4,359	44	3,258	33	0.98 (0.93–1.03)	0.87 (0.81–0.94)	1.03 (0.95–1.11)
2014–2015	51,842	39,267	76	12,575	32	10,006	79	5,651	45	4,320	34	0.89 (0.84–0.94)	0.75 (0.70–0.82)	0.93 (0.86–0.99)

*Cohort A: antidepressant treatment two or more years before childbirth, Cohort B: antidepressant treatment between one to two years before childbirth, Cohort C: antidepressant treatment in year before childbirth

** Percentage of all individuals prescribed antidepressants

LR Chi2 = 494.08, *p* < 0.0001

^†^Models adjusted for age, year of prescription and social deprivation

Regarding the timing of antidepressant treatment **before** childbirth, 18% (37,161/202,303) had at least one prescription two or more years prior, 10% (19,967/202,303) received at least one prescription between one and two years before, and 7% (14,363/202,303) were prescribed antidepressants in the year leading up to childbirth. Of the women who received antidepressant treatment 80% or more have received treatment two or more years before childbirth, while one in three of the recipients had at least one prescription in the year before childbirth (Table 1).

### Association between history of antidepressant treatment and postnatal treatment

Of the women with **no** history of antidepressant treatment 10,258/155,824 (7%) received antidepressants after childbirth. Women prescribed antidepressants before childbirth were 8 times more likely to receive an antidepressant in the year after childbirth (aOR: 8.21, 95% CI: 7.89–8.55 vs. reference group with no history) (Table 2). An antidepressant prescription in the year before childbirth conferred a 17 times increased likelihood for receiving postnatal antidepressant (aOR: 17.77 (16.87–18.73) (Table 2). Unadjusted values are provided in Supplementary Table C.

**Table 2:** Proportion and odds of women being issued an antidepressant prescription after childbirth by previous history

Time period	Received an antidepressant treatment prior to childbirth *n* (% down)		Received an antidepressant treatment in the year after childbirth *n* (% across)	Odds of post-partum antidepressant treatment adjusted^†^ OR, 95% CI
Study entry to childbirth	No	155,824 (77.0)	10,258 (6.6)	1
	Yes	46,479 (23.0)	16,577 (35.7)	8.21 (7.89–8.55)
Cohort A: At least one prescription between study entry to 2 years before childbirth	Yes	37,161 (80.0)	4,139 (18.4)	5.95 (5.71–6.20)
Cohort B: At least one antidepressant prescription between 2 years and 1 year before childbirth	Yes	19,967 (43.0)	3,206 (33.3)	11.40 (10.89–11.95)
Cohort C: At least one1 year before childbirth to childbirth	Yes	14,363 (7.1)	9,232 (64.3)	17.77 (16.87–18.73)

^†^Adjusted for maternal age, social deprivation and calendar year

The likelihood of receiving an antidepressant prescription in the year following childbirth was highest for women under the age of 30 (Table 1). Similar trends were noted when stratifying by previous history of antidepressant treatment, with the most pronounced effects seen in those without a prior history (aOR: 3.94, 95% CI: 3.59–4.31 for ages 15–19 years vs. aOR: 0.73, 95% CI: 0.45–1.18 for ages 45–49 years). The likelihood of receiving an antidepressant prescription in the year after childbirth increased with deprivation across all groups (Table 1).

### Patterns of antidepressant treatment in the perinatal period

**Fig. 1:**
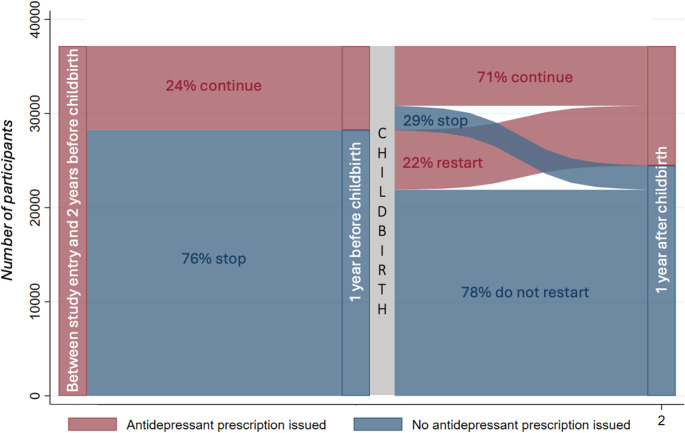
Sankey diagram showing continuation or discontinuation of antidepressant prescriptions at different time points across the perinatal pathway for women in Cohort A- those issued most recent antidepressant prescription at least 2 years before childbirth

The number of women receiving treatment at multiple time points is detailed in Supplementary Table B. Among the 37,161 women who received an antidepressant prescription at least two years before childbirth (cohort A), 28,242 (76%) did not have a prescription in the year before childbirth. However, of these, who discontinued treatment 6,350 (22%) resumed their antidepressant prescription in the year after childbirth (Fig. 1).

**Fig. 2:**
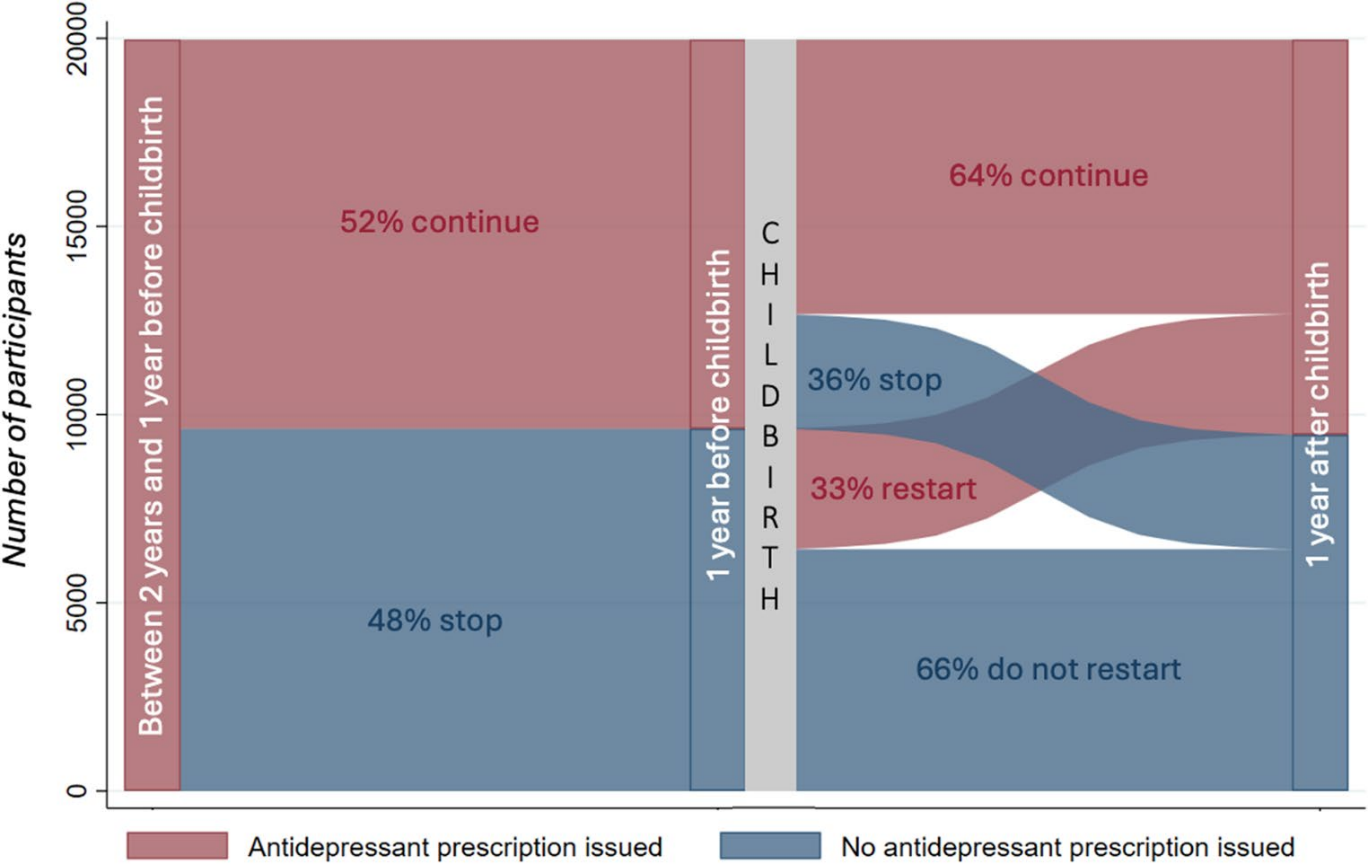
Sankey diagram showing continuation or discontinuation of antidepressant prescriptions at different time points across the perinatal pathway for women in Cohort B - those issued an antidepressant prescription between 2 and 1 years before childbirth

Overall, it is a relatively small proportion of the women who received antidepressant treatment throughout the perinatal period (6,341/37,161 (17%)) (Fig. 2). Thus, among the 37,161 women who received an antidepressant prescription at least two years before childbirth (Cohort A), 8,919 (24%) maintained their treatment throughout conception and pregnancy, with 6,341 (71%) of these women also receiving a prescription in the year after childbirth (Fig. 2).

Of the 19,967 women who were treated between two and one year prior to childbirth (cohort B), 9,627 (48%) did not have a prescription in the year before childbirth, yet 3,206 (33%) of those who discontinued just before or during pregnancy resumed treatment in the year after childbirth (Fig. 2). In total, 10,500 (53%) of the 19,967 women who received antidepressant prescriptions between two and one year prior to childbirth also received treatment in the year after childbirth.

## Discussion

### Main findings

In this study, we found substantial movement in and out of antidepressant treatment before, during and after pregnancy. Among women with a previous prescription, around half discontinued treatment during preconception and pregnancy, yet a third of these women restarted treatment in the year after childbirth. Initiation of treatment after childbirth was less common among women with no prior antidepressant history, with 7% receiving a prescription in the year following childbirth. Postnatal treatment was most common among those with more recent use: 64% of women who received antidepressants during conception/pregnancy also received treatment in the year after childbirth.

### Strengths and weaknesses of the study

A significant strength of this study is its size; it is one of the largest prospective investigations into antidepressant use surrounding childbirth to date. By utilising EHRs, the study captures real-world data, allowing for longitudinal analysis of prescribing trends that reflect clinical practice in the UK. The ability to stratify prior antidepressant use into different time periods around pregnancy and childbirth provides valuable insights into postnatal prescription likelihood, aiding GPs in identifying women who may need treatment after childbirth.

However, using antidepressant prescriptions as a proxy may underestimate the prevalence of perinatal depression, as it excludes women who are untreated or who experience milder depressive episodes. It should be noted that, while largely prescribed to manage depression, antidepressants can also be prescribed for other indications, such as anxiety, but this is challenging to identify in EHRs as reasons for a prescription are often not captured. Furthermore, EHR data only indicate prescriptions issued, without confirming whether women filled or adhered to these prescriptions. Lastly, we chose to group pregnancy and preconception into one exposure category ‘one year before childbirth’ to provide a consistent time frame to analyse across all women regardless of pregnancy length, future research could further stratify this time window.

### Possible explanations of findings and clinical implications

Our finding that around half of women on antidepressants discontinue treatment during conception and pregnancy aligns with previous studies (Trinh et al. 2023). Our findings that among those who stopped, a third resumed treatment post-partum, is also consistent with smaller studies (Trinh et al. 2022). However, our study is one of the first to document that 53% of women treated before conception also received treatment in the year following childbirth. Clinically, the decision to continue or discontinue antidepressants during pregnancy should be individualised, taking into account personal risk factors and patient preferences. This study provides evidence to support informed discussions about the potential need for antidepressants after childbirth, especially given that stopping treatment may increase the risk of relapse.

While some mothers are concerned about medication safety during pregnancy (Sanders et al. 2023), it is essential to provide evidence on the relative risks of antidepressants, emphasising that the absolute risk of adverse events remains small (Desaunay et al. 2023). It is also important to highlight that no conclusive evidence supports the benefit of preventive cognitive therapy for women discontinuing antidepressants during pregnancy (Molenaar et al. 2020). Clinicians should engage in shared decision-making, regularly review prescriptions during the perinatal period, and prescribe the lowest effective dose to minimise potential harm.

Our findings corroborate previous research indicating that a history of depression increases the risk of postnatal depression (Al-abri et al. 2023, Davé et al. 2010, Boyce 2003, Silverman et al. 2017). While retrospective studies have suggested that depression during pregnancy carries the highest risk for postnatal depression (Johansen et al. 2020), our study adds to the literature by providing data on antidepressant prescribing stratified before, during and after pregnancy in a large prospective cohort. It is crucial to recognise that any history of antidepressant treatment heightens the risk of postnatal prescribing.

Additionally, our findings demonstrate that younger maternal age and increased socioeconomic deprivation are associated with a higher likelihood of postnatal antidepressant treatment as found by others (Katon et al. 2014, Petersen et al. 2018). These insights can help identify high-risk individuals who previously required antidepressants, ensuring they receive appropriate screening, early intervention, and closer monitoring both during pregnancy and after childbirth. Implementing alerts in EHRs could facilitate reviews of high-risk individuals during routine postnatal check-ups (Smith et al. 2020). Raising awareness of individual risk factors can also empower patients and families to recognise symptoms and seek help when needed.

There remains ongoing debate about whether postnatal depression represents a new condition or a continuation of previous depression, with emerging evidence highlighting differences in symptomatology, epigenetics, and treatment responses (Batt et al. 2020). Our study found that 7% of women with no prior antidepressant prescriptions initiated treatment in the year following childbirth, more than double the baseline incidence (3%) in a similar age cohort (18–39 years), suggesting that the postnatal period as a whole is a vulnerable period for new onset mental health conditions (McCrea et al. 2016). Clinicians must remain vigilant, using standard screening questions to identify women who may develop postnatal depression, even in those without a prior history of depression.

### Unanswered questions and future research

The findings from this study can guide further research, such as developing validated risk scores or algorithms to identify women at high risk for perinatal depression, particularly those with no prior history. Future studies should explore differences between de novo postnatal depression and ongoing depression, with a focus on identifying relevant risk factors. This could inform tailored screening methods or treatment options for different groups. Additionally, research should investigate the reasons for antidepressant discontinuation and ways to effectively communicate the risks and benefits of ongoing treatment during pregnancy.

## Conclusion

Approximately half of the women who are treated with antidepressants in the year before pregnancy discontinued treatment during conception and pregnancy, but one in three of those who stopped resumed treatment after childbirth. These findings can enhance patient counselling by providing information about similar cases, supporting informed decisions about continuing or discontinuing antidepressants during pregnancy. Furthermore, 7% of women who had never received an antidepressant treatment before childbirth, initiated treatment in the year after childbirth, with younger women being the most likely. This suggests that the postnatal period can be an important time for identifying mental health needs for all women. A history of antidepressant prescriptions prior to childbirth was strongly associated with subsequent prescriptions, with more recent prescriptions correlating with higher likelihood of treatment in the year following birth. Identifying high-risk women using these factors can facilitate increased support and earlier recognition of symptoms.

## Supplementary Information

Below is the link to the electronic supplementary material.

ESM 1 (DOCX 77.7 KB)
