# Supplementary material for: Continuation and discontinuation of antidepressant treatment before, during and after pregnancy: a cohort study
Source: Arch Womens Ment Health. 2026 Mar 10;29(2):49. doi: 10.1007/s00737-026-01683-5 (PMC12971859; doi:10.1007/s00737-026-01683-5)
Supplement: Supplementary file 1 — (DOCX 77.7 KB) [file 737_2026_1683_MOESM1_ESM.docx]

**Supplementary Material**

**
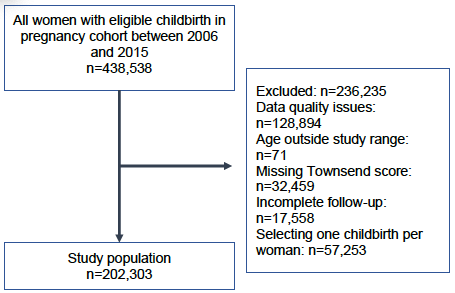
**

**Supplementary Figure A:** Flow diagram showing application of study inclusion and exclusion criteria

*Data quality issues were applied whereby practices which did not have acceptable computer use (ACU) or acceptable mortality rates (AMR) by the date of childbirth were excluded. ACU is the date a

practice was continuously entering on average at least two therapy records, one medical record and one additional health data record per patient per year; and AMR is the date a practice has comparable mortality rates to the rest of the UK, given the size and demographics of the practice. Women who had been registered at a practice for less than 6months were also excluded.

**Supplementary Table B:** Overlap between cohort definitions: Proportion of women meeting multiple eligibility criteria*

|  |  | **Total number of women with prescriptions** | **How many women receive an antidepressant prescription in this time period (% across)** | | | |
| --- | --- | --- | --- | --- | --- | --- |
|  |  |  | **A** | **B** | **C** | **D** |
| **Of those receiving an antidepressant prescription in this time period.** | **A** | 37,161 |  | 13,197 (36%) | 8,919  (24%) | 12,691 (34%) |
|  | **B** | 19,967 | 13,197 (66%) |  | 10,340 (52%) | 10,500 (53%) |
|  | **C** | 14,363 | 8,919  (62%) | 10,340 (72%) |  | 9,232  (64%) |
|  | **D** | 26,835 | 12,691 (47%) | 10,500 (39%) | 9,232  (34%) |  |

*Time period A: at least 2 years before childbirth*

*Time period B: between 2 years and 1 year before childbirth*

*Time period C: 1 year before childbirth to childbirth*

*Time period D: 1 year after childbirth*

*This table shows the degree of overlap between cohort definitions. Each row represents women assigned to a given cohort, and the values indicate the proportion of these women who also meet the definition for each of the other cohorts. For example, of the 37,161 who meet the criteria for cohort A, 36% (13,197) also meet the criteria for cohort B.

**Supplementary Table C:** Odds of receiving a post-partum antidepressant prescription based on antidepressant exposure prior to childbirth

| **Characteristic** | **Odds of post-partum antidepressant treatment if no history of antidepressant treatment before childbirth** | | **Odds of post-partum antidepressant treatment if history of antidepressant treatment before childbirth** | |
| --- | --- | --- | --- | --- |
|  | Unadjusted (OR, 95% CI) | Adjusted* (OR, 95% CI) | Unadjusted (OR, 95% CI) | Adjusted* (OR, 95% CI) |
| Maternal age | | | | |
| 15-19 | 4.22 (3.90-4.57) | 3.94 (3.59-4.31) | 1.68 (1.42-1.99) | 1.64 (1.36-1.97) |
| 20-24 | 2.59 (2.44-2.74) | 2.46 (2.30-2.63) | 1.21 (1.144-1.29) | 1.18 (1.12-1.36) |
| 25-29 | 1.38 (1.31-1.46) | 1.35 (1.27-1.43) | 1.03 (0.98-1.09) | 1.02 (0.97-1.07) |
| 30-34 | 1 | 1 | 1 | 1 |
| 35-39 | 0.89 (0.84-0.95) | 0.90 (0.84-0.96) | 1.00 (0.95-1.09) | 1.01 (0.95-1.06) |
| 40-44 | 0.88 (0.79-0.99) | 0.89 (0.80-0.99) | 0.96 (0.89-1.05) | 0.97 (0.89-1.06) |
| 45-49 | 0.72 (0.44-1.17) | 0.73 (0.45-1.18) | 0.81 (0.59-1.11) | 0.81 (0.59-1.11) |
| Townsend Score quintile | | | | |
| 1 (least deprived) | 1 | 1 | 1 | 1 |
| 2 | 1.14 (1.06-1.22) | 1.08 (1.01-1.15) | 1.06 (0.99-1.13) | 1.05 (0.98-1.13) |
| 3 | 1.28 (1.20-1.37) | 1.12 (1.03-1.20) | 1.07 (1.01-1.14) | 1.06 (0.99-1.13) |
| 4 | 1.53 (1.44-1.63) | 1.20 (1.11-1.31) | 1.14 (1.07-1.21) | 1.11 (1.03-1.19) |
| 5 (most deprived) | 1.82 (1.71-1.95) | 1.28 (1.16-1.42) | 1.24 (1.17-1.32) | 1.20 (1.12-1.29) |
| Year group of childbirth | | | | |
| 2006-2007 | 1 | 1 | 1 | 1 |
| 2008-2009 | 0.93 (0.87-1.00) | 0.91 (0.84-0.98) | 0.95 (0.88-1.02) | 0.95 (0.88-1.02) |
| 2010-2011 | 0.98 (0.91-1.05) | 0.93 (0.86-1.00) | 1.00 (0.93-1.07) | 1.00 (0.93-1.07) |
| 2012-2013 | 0.90 (0.84-0.97) | 0.87 (0.81-0.94) | 1.03 (0.96-1.11) | 1.03 (0.95-1.11) |
| 2014-2015 | 0.77 (0.72-0.83) | 0.75 (0.70-0.82) | 0.93 (0.87-1.00) | 0.93 (0.86-0.99) |
